# The biomechanical study of the influence to the forefoot plantar pressure of the first tarsometatarsal joint fracture-dislocation fixed by three different implants

**DOI:** 10.12669/pjms.331.11908

**Published:** 2017

**Authors:** Xiao Yu, Qing-jiang Pang, Xian-jun Chen

**Affiliations:** 1 Dr. Xiao Yu, PhD. Department of Orthopedics, Ningbo NO.2 Hospital, Ningbo, 315010, China; 2 Dr. Qing-jiang Pang, PhD. Department of Orthopedics, Ningbo NO.2 Hospital, Ningbo, 315010, China; 3 Dr. Xian-jun Chen, MD. Department of Orthopedics, Ningbo NO.2 Hospital, Ningbo, 315010, China

**Keywords:** Tarsometatarsal joint, Fracture dislocation, Implant, Plantar pressure, Biomechanics

## Abstract

**Objectives:**

To study the influence of forefoot plantar pressure of the first tarsometatarsal joint fracture-dislocation by three different implants to provide experimental reference in selecting implants.

**Methods:**

Eight fresh foot specimens were made into the models of the first tarsometatarsal joint fracture-dislocation, which were fixed with 3.5 mm cortical screw, 1/4 tubular plate and compressive staple in turn. After the loading of 600N, the changes of the plantar pressure in forefoot were measured by the method of the F-scan plantar pressure system.

**Results:**

After first tarsometatarsal joint fracture-dislocation, the peak pressure under the first metatarsal head would decrease, while the pressure under the second metatarsal head would increase(*P*<0.05). When the first tarsometatarsal joint was fixed with screw or plate respectively; the peak pressure under the two metatarsal heads would tend to be normal. However, the staple fixation would show the statistical significant difference, although the peak pressure under the first and second metatarsal heads were recovered in some extent(*P*<0.05).

**Conclusions:**

After the first tarsometatarsal joint fracture-dislocation, the plantar pressure might be compensated partly by the adjacent metatarsal heads. While the first tarsometatarsal joint fracture-dislocation was fixed by screw or plate, the plantar pressure of the forefoot would return to the normal state. However, if the joint was fixed by the staple, it would still be difficult to return the plantar pressure to be normal.

## INTRODUCTION

The first tarsometatarsal (TMT) joint is an important part of the medial column of the foot, whose integrity has important significance in the maintenance of foot arch and load transfer. The first TMT joint injury should be treated actively to recover the alignment of the midfoot and to ensure the load transfer from forefoot to the midfoot.^1^ Therefore, the implants choice demand is very high, especially in the high energy injury cases with multiple tarsometatarsal joints fracture-dislocation. Once improper implants were chosen, it is easy to cause the complications such as implant breakage, loss of reduction and malunion.^2^

Although a variety of implants can fix the TMT joint, such as screw and plate, staple and Endobutton, the indications of each implant have not formed a consensus. Furthermore, most doctors only pay attention to the integrity of fracture and articular surface and rarely notice if the implant causes the abnormal plantar pressure, which is also an important reason for the failure of surgery. In fact, some areas of the anatomical region of the foot support most of the body’s weight and regulate the body’s balance. Once the injuries of these areas occur, it may appear the disorder of gravity support and balance regulation. At this point, if the plantar pressure can be measured in these areas, it is helpful for us to understand the pathophysiology characteristics of the damaged area and even to the whole foot and ankle, which can provide important reference value for the subsequent treatment.^3^

## METHODS

### Study Subjects

Eight fresh foot specimens were chosen for this study (Provided by Ningbo University, School of medicine). All the specimens have the normal shape without toe defect or muscle, ligament, tendon injury. By X-ray examination, degenerative disease, fracture, tumor, structural malformation disease were ruled out.

### Experimental equipment

2T torsion load testing machine(Changchun Mechanical Science Research Institute Co., Ltd.), F-Scan^®^ insole plantar pressure analysis system (Tekscan Co., Ltd, USA), 6-hole 1/4 tubular plate (KangHui Co., Ltd), Ø3.5mm Fully threaded cortical screw (Synthes Co., Ltd, Switzerland), CHARLOTTE™ Compressed staple(Wright Co., Ltd, USA).

### Experimental methods

### Specimen preparation

**Fig. 1:**
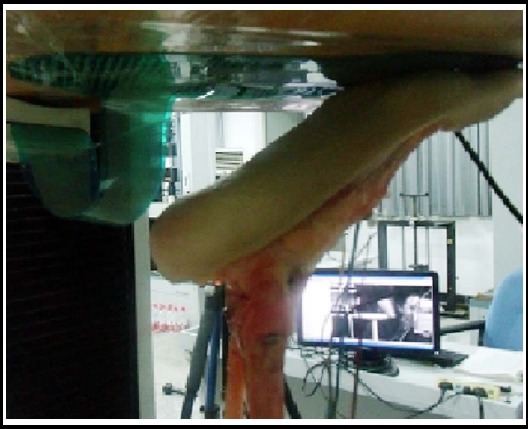
The specimen was fixation at the 30° of ankle plantar flexion, the sole transducer could only be contacted with the forefoot.

The dorsum skin, subcutaneous tissue and muscle of the specimens was excised to expose the medial cuneiform, intermediate cuneiform, 1^st^ and 2^nd^ metatarsal bone and the dorsal ligament between the medial cuneiform and the base of 1^st^ metatarsal. The ankle joint was fixed in plantar flexion of 30° and the specimen was fixed on the base of the torsion load testing machine. Suitable insole sensor was selected according to the size of the specimen. The position of the specimen was adjusted to ensure that the insole sensor was only in contact with the forefoot (Fig.1).

### Working condition setting

**Fig. 2:**
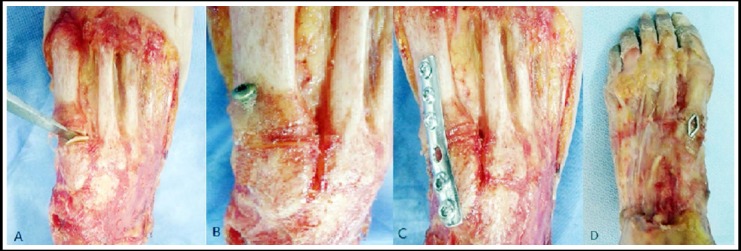
The establishment of the fracture and dislocation model of the first tarsometatarsal joint by osteotomy and cutting the ligaments (A). During the study, the model was fixed by screw (B), plate (C) and staple (D) separately.

Five working conditions were set to test the specimens. Condition 1: the intact state of the bone-ligament structure. Condition 2: the fracture-dislocation of the first TMT joint. Condition 3: The first TMT joint was fixed by a fully threaded cortical screw from the base of the first metatarsal bone to the medial cuneiform. Condition 4: The first TMT joint was fixed by a 6-hole 1/4 tubular plate. Condition 5: The first TMT joint was fixed by a compressed staple (Fig.2). The model establishment of first TMT joint fracture-dislocation was referred to the Alberta method to cut off of the ligaments between the medial cuneiform and the first metatarsal then do osteotomy along the articular surface to result in the intra-articular fracture-dislocation model of the first TMT joint.^4^

**Fig. 3:**
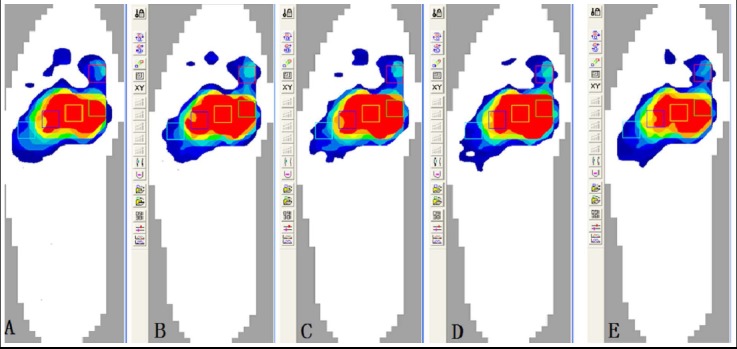
The plantar pressure distribution in forefoot at 600N loading, the color from red to yellow, green, light blue to deep blue demonstrated the plantar pressure from large to small. A. The plantar pressure distribution in the normal state. B. In the state of the first TMT joint injury. C. In the state of screw fixation. D. In the state of plate fixation. E. In the state of staple fixation.

### Data collection

The software of F-scan mobile Clinical 5.26.5 was started before loading to display the real-time monitoring image of the plantar pressure and complete the correction of the system. The load was from 0N to 600N uniformly in each working condition. The plantar pressure images of 600N were extracted and the plantar pressure was calculated through the software of F-scan mobile Clinical 5.26.5. When a working condition was finished loading and data acquisition, the specimens are processed under the next condition until all the working conditions were completed.

### Statistical analysis

The peak pressure of each test site under 600N load was recorded by mean±standard deviation (±s). The statistical software SPSS 13.0 was used for data analysis, the test level of α=0.05. The variance analysis was used to compare the peak pressure of each test site in each working condition.

## RESULTS

The peak plantar pressure of the test sites under 600N is shown in Table-I. The variance analysis showed no significant difference was found of the peak pressure in hallux group, the third and fourth metatarsal heads group and the fifth metatarsal head group (*P*>0.05), however, significant difference of the peak pressure was found in the first metatarsal head group and the second metatarsal group (*P*<0.05).

**Table-I:** The peak plantar pressure in each test site at the loading to 600N (±s, Kpa, n=8).

	Hallux	1^st^ MH	2^nd^ MH	3^rd^ and 4^th^ MHs	5^th^ MH
Condition 1	64.32±11.44	140.88±13.21	216.47±15.04	177.74±12.84	69.47±10.82
Condition 2	72.57±11.67	118.08±12.30	242.57±14.33	179.34±12.41	68.27±11.84
Condition 3	63.75±12.16	141.78±10.62	216.14±11.15	181.31±13.66	72.48±11.63
Condition 4	63.50±12.42	140.56±10.40	216.32±10.55	184.78±12.97	70.21±10.57
Condition 5	68.84±10.04	124.37±11.04	236.37±14.17	182.25±13.77	71.08±12.86
F	0.746	10.908	5.729	0.254	0.144
P	0.570	0.000	0.002	0.904	0.976

When the first TMT joint was injured (condition 2), the peak pressure in the first MH would decrease while the peak pressure in the second MH would increase compared with condition 1 (*P*<0.05).

When the first TMT joint was fixed by screw (condition 3) and plate (condition 4) separately, the peak pressure in the first MH would increase while the peak pressure in the second MH would decrease compared with condition 2 (*P*<0.05). However, there were no significant differences compared the condition 3 with the condition 4 of the peak pressure in the first MH and the second MH(*P*<0.05).

When the first TMT joint was fixed by staple (condition 5), although the peak pressure in the first MH would increase, it was still less than the peak pressure in condition 1 (*P*<0.05). On the contrary, although the peak pressure in the second MH would decrease, it was still more than the peak pressure in condition 1 (*P*<0.05). While compared the condition 5 with the condition 3 and condition 4, peak pressure in the first MH in condition 5 is less than condition 3 and condition 4, however, the peak pressure in the second MH in condition 5 is more than condition 3 and condition 4(*P*<0.05).

## DISCUSSION

### The model establishment of the first TMT joint fracture-dislocation

The direction of the first TMT joint dislocation can be divided into dorsal dislocation and plantar dislocation in the cross section. In clinical, dorsal dislocation is more common than plantar dislocation.^5^ Furthermore, midfoot injury often associated with ligaments injury and fracture in other parts of the foot, but the present experimental conditions cannot completely simulate specific parts of the fracture dislocation and ligament injury according to the injury mechanism. Therefore, in this study, the model of the first TMT joint fracture-dislocation could only be established by cutting the ligaments in the first TMT joint and doing the osteotomy in the base of the first metatarsal. This model could be classified as Type B1 of Myerson classification. In the normal gait cycle, the load of the TMT joint increased gradually from initial contact to terminal stance and it reached the maximum in the pre-swing. At this time, the ankle was 15° plantar flexion. However, if the gait was larger, the plantar flexion range would increase. The TMT joint was easy to be injured when the load was axially loaded to the ankle in an extreme plantar flexion state. Therefore, in this study the specimen was axially loaded in 30° plantar flexion of the ankle with the maximum load of 600N (body weight of a normal adult).^6^ Actually, in gait cycle, the TMT joint bears more than 600N, however, when the load exceeded 600N, the fixation of ankle in 30°plantar flexion would easily failed.

### The changes of forefoot plantar pressure of the first TMT joint fracture-dislocation

Plantar pressure measurement is a clinical detection technology to measure the static or dynamic plantar pressure to reveal the characteristics of plantar pressure distribution, which could provide references to analyzed the etiology, progression and judge the prognosis. Schepers^7^ studied the gait and plantar pressure of 26 patients of TMT joints injury and the results showed that compared with the contralateral foot, the contact area and contact time of the affected forefoot to the ground was reduced, while the contact area and the peak pressure of midfoot increased. If this state could not be corrected in a long-term, it may become one of the causes of pain in midfoot. In this study, we first measured the forefoot plantar pressure of 600N before and after the first TMT joint injury. The results showed the injury of the integrity of the first TMT joint would cause the decrease of the peak pressure in the first MH and increase of the peak pressure in the second MH. We hypothesize that there might be a regulating mechanism of “load transfer” in plantar pressure of the forefoot. In normal condition, the plantar pressure in the first MH can be regulated by the fat pad and the plantar fascia.^8^ However, the first TMT joint instability would affect the activity of the first metatarsal and the self-regulating mechanism might be impaired. The contact area of first MH to the ground would be increased and the buffer capacity of first MH to the plantar pressure would be reduced. The reduced part might need to be compensated by the adjacent hallux and the second MH. Although this kind of “load transfer” could relieve the plantar pressure in the injury of TMT joint to some extent, the additional increased plantar pressure in other part of the forefoot might cause the complications such as the forefoot metatarsalgia, painful callosities, osteonecrosis of the MH, plantar fasciitis and pressure ulcer if it existed in a long time.^9^,^10^

### The influence to the forefoot plantar pressure of the first TMT joint fracture-dislocation fixed by three different implants

Currently, it is emphasized that in the process of the fracture reduction or the correction of the complicated foot deformity, the restoration of the plantar pressure balance is more important than the foot shape recovery.^11^ Schepers^12^ studied the plantar pressure in 21 patients of intra-articular calcaneal fracture and concluded that there was no direct relationship between the plantar pressure distribution and the clinical effect. Therefore, the authors believed that the clinical evaluation cannot completely replace the plantar pressure analysis. At present, the plantar pressure analysis is often used to determine the appropriate implants and evaluation of the surgical efficacy.^13^ In this study, the results showed that the screw or plate fixation to the first TMT joint could make the peak pressure in the first MH and the second MH tend to be normal. There was no significant difference in the ability of the plate and screw to restore the peak pressure to be normal in the first MH and the second MH. When the first TMT joint was fixed by the staple, although the peak pressure would decrease in the second MH and increase in the first MH, significant differences could still be found compared with the normal state and the fixation by screw and plate. The results illustrated that when the stability of the first TMT joint was restored, the additional load born by hallux and the second metatarsal would be redistributed again to the first MH by the “load transfer” mechanism, the forefoot plantar pressure will therefore be balanced. It suggests that when the first TMT joint is fracture-dislocation, the accurate anatomical reduction and internal fixation by screw or plate helps to maintain the balance of the plantar pressure to avoid foot disease. As for the staple, we speculated that the staple fixation in complete dislocation of TMT joint (both dorsal and plantar ligaments injury) may cause fixation failure because of the less pullout resistance of the staple in this type of injury. Linked to this study, if the specimen of first TMT joint fracture-dislocation was fixed by staple, the stability of the first TMT joint could only be recovered partly. These results suggested that attention should be paid to indications of the clinical application of staple.
